# Null mutation for Macrophage Migration Inhibitory Factor (MIF) is associated with less aggressive bladder cancer in mice

**DOI:** 10.1186/1471-2407-7-135

**Published:** 2007-07-24

**Authors:** John A Taylor, George A Kuchel, Poornima Hegde, Olga S Voznesensky, Kevin Claffey, John Tsimikas, Lin Leng, Richard Bucala, Carol Pilbeam

**Affiliations:** 1 Division of Urology, University of Connecticut Health Center, Farmington, CT, USA; 2 Department of Medicine, University of Connecticut Health Center, Farmington, CT, USA; 3 Department of Pathology, University of Connecticut Health Center, Farmington, CT, USA; 4 Department of Vascular Biology, University of Connecticut Health Center, Farmington, CT, USA; 5 Division of Epidemiology and Statistics, University of Connecticut Health Center, Farmington, CT, USA; 6 Department of Medicine & Pathology, Yale University, New Haven, CT, USA

## Abstract

**Background:**

Inflammatory cytokines may promote tumorigenesis. Macrophage migration inhibitory factor (MIF) is a proinflammatory cytokine with regulatory properties over tumor suppressor proteins involved in bladder cancer. We studied the development of bladder cancer in wild type (WT) and MIF knockout (KO) mice given N-butyl-N-(4-hydroxybutyl)-nitrosamine (BBN), a known carcinogen, to determine the role of MIF in bladder cancer initiation and progression.

**Methods:**

5-month old male C57Bl/6 MIF WT and KO mice were treated with and without BBN. Animals were sacrificed at intervals up to 23 weeks of treatment. Bladder tumor stage and grade were evaluated by H&E. Immunohistochemical (IHC) analysis was performed for MIF and platelet/endothelial cell adhesion molecule 1 (PECAM-1), a measure of vascularization. MIF mRNA was analyzed by quantitative real-time polymerase chain reaction.

**Results:**

Poorly differentiated carcinoma developed in all BBN treated mice by week 20. MIF WT animals developed T2 disease, while KO animals developed only T1 disease. MIF IHC revealed predominantly urothelial cytoplasmic staining in the WT control animals and a shift toward nuclear staining in WT BBN treated animals. MIF mRNA levels were 3-fold higher in BBN treated animals relative to controls when invasive cancer was present. PECAM-1 staining revealed significantly more stromal vessels in the tumors in WT animals when compared to KOs.

**Conclusion:**

Muscle invasive bladder cancer with increased stromal vascularity was associated with increased MIF mRNA levels and nuclear redistribution. Consistently lower stage tumors were seen in MIF KO compared to WT mice. These data suggest that MIF may play a role in the progression to invasive bladder cancer.

## Background

Bladder cancer is a common urinary tract malignancy. In 2007 there will be an estimated 67,160 new cases with 13,750 deaths in the United States alone [1]. Stage at diagnosis is fundamental to outcome as 50% of patients with muscle invasion have metastatic disease. Current therapies for advanced disease are disappointing. Even with aggressive surgical and medical treatment most patients with advanced bladder cancer ultimately succumb to their disease.

Recent research has suggested an important role for inflammatory cytokines and chemokines in the development of cancer. They may promote tumorigenesis by providing an environment that enhances cell proliferation, survival and migration. Macrophage migration inhibitory factor (MIF) is a proinflammatory cytokine that has regulatory properties over mediators such as p53 and the retinoblastoma protein (pRb) known to be involved in invasive bladder cancer [2,3]. Overexpression of MIF has been implicated in several cancers [4-6]. We studied the effects of knocking out the gene for MIF (*mif*-/-) on the development of bladder cancer in mice given N-butyl-N-(4-hydroxybutyl)-nitrosamine (BBN).

## Methods

Mice lacking MIF (MIF-KO) were generated by homologous recombination and backcrossed into a pure C57BL/6 background (generation N8) [7]. Animals were bred and maintained at the University of Connecticut Health Center for Laboratory Animal Care under National Institutes of Health guidelines. All procedures were approved by an institutional animal care committee. Fifty 5 month old C57Bl/6 MIF-KO and WT mice were housed in a controlled environment with a 12 hour light – 12 hour dark cycle and provided food and water ad libitum. The experimental group received BBN (TCI America, Portland, OR) 0.05% in water in brown bottles throughout. Two mice from each group (WT control and treated, KO control and treated) were euthanized by CO_2 _inhalation at 4, 8, 12, 16, 20 and 23 weeks. Bladders were harvested and inspected for tumor prevalence. Half of each bladder, and tumor if present, was used for pathologic evaluation and half for analysis of mRNA.

### Pathologic analysis

Bladder halves were placed in PBS/formaldehyde for 24 hours and then transferred to PBS. Bladder halves were sagitally sectioned into 3 levels (inner, mid, outer) and stained with H&E. Characteristics such as number of cell layers, nuclear size, nuclear membrane irregularity, chromatin pattern, nuclear:cytoplasmic ratio, presence of nucleoli and mitosis were evaluated in order to provide a final diagnosis of (1) normal, (2) metaplasia, (3) atypia, (4) carcinoma in situ (CIS) and (5) carcinoma. The term carcinoma is applied to all cancers as lesions showed mixed histology with both transitional and squamous elements. Tumor stage and grade are the most predictive variables with regard to prognosis and these were the main outcome metrics in our model. Invasion was diagnosed if tumor cells were seen infiltrating the muscle layers of the bladder. A single pathologist (P.H.) reviewed all slides in a blinded fashion.

### Immunohistochemistry (IHC)

IHC for MIF was performed in a standard method. Endogenous peroxidase activity was blocked by treatment with 3% H_2_O_2 _in PBS for 30 min. Non-specific staining was blocked by incubation with Power Block™ Universal Blocking agent (Biogenex, San Ramon, CA). Specimens were rinsed in PBS. Tissue sections were stained with a rabbit polyclonal anti-MIF antibody (Cell Sciences, CPM300). Sections were incubated overnight at 4–8°C (dilution 1:1000). Bound antibody was detected with a secondary biotinylated anti-rabbit antibody for 30 min at room temperature (1:200) and visualized using diaminobenzidine (DAB) as a chromogenic substrate according to the manufacturer's instructions (Vector Labs, Burlingame, CA).

Sections were evaluated for degree of nuclear staining and scored from 0–3 corresponding to negative, weak, intermediate and strong staining respectively. Results were quantified using an H-score as previously described [8]. Three fields were chosen representing areas of maximal staining at 200× magnification. The total number of nuclei and total number staining at each intensity were counted. The average percentage positive for each group was calculated. H-score was calculated as equal to (% of cells stained at intensity 0 × 0) + (% of cells stained at intensity 1 × 1) + (% of cells stained at intensity 2 × 2) + (% of cells stained at intensity 3 × 3). This resulted in a score between 0 and 300 where 300 is equal to 100% of tumor cell nuclei staining strongly.

IHC for PECAM-1 was performed in a standard method. Endogenous peroxidase activity was blocked by treatment with 3% H2O2 for 10 min. Non-specific staining was blocked by incubation with Power Block™ for 10 min. Tissue sections were stained with a goat polyclonal anti-PECAM antibody (Santa Cruz Biotechnology, M20 SC-1506) at 1:500 dilution in PBS-BSA overnight at 4°C. Bound anti-PECAM-1 antibody was detected with a secondary biotinylated horse-anti-goat antibody for 30 min at room temperature (1:200) and visualized using DAB.

Microvessel density (MVD) was calculated by the Weidner method [9]. Using light microscopy (200× field), tumors were evaluated for associated angiogenesis. The region in each specimen that represented the area of maximal activity was then processed to capture the vascular structures present with minimal background using Image Pro Plus analytical software (Media Cybernetics, Silver Spring, MD). Results were quantified and defined in terms of pixels/field.

### Quantitative polymerase chain reaction for mRNA

Quantitative real time PCR (qPCR) was performed for MIF and TNF-α mRNA with GAPDH as an internal control. Total RNA was extracted from bladder tissues with TRIzol^® ^Reagent (Invitrogen Life Technologies, Carlsbad, CA) following the manufacturer's protocol. Total RNA was converted to cDNA by ABI High Capacity cDNA Archive Kit (Applied Biosystems, Foster City, CA) following the manufacturer's protocol. qPCR was performed for different gene expression in separate wells (singleplex assay) of a 96-well plate in a reaction volume of 20 μl. Three replicates of each sample were amplified using Assays-on-Demand Gene Expression assay (Assay # Mm01604696_g1 for MIF, Mm00443258_m1 for TNF and Mm99999915_g1 for GAPDH) which contain predesigned, unlabeled gene-specific PCR primers and TaqMan MGB FAM dye-labeled probe. The PCR reaction mixture (including 2 × TaqMan Universal PCR Master Mix, 20 × Assays-on-Demand Gene Expression Assay Mix, 50 ng of cDNA) was run in Applied Biosystems ABI Prism 7300 Sequence Detection System instrument utilizing universal thermal cycling parameters. For genes for which the efficiencies of target and endogenous control amplification were approximately equal, the relative quantification expression in a test sample to a reference calibrator sample (ΔΔCt Method) was used for data analysis. For genes which were not amplified with the same efficiency as the endogenous control, The Relative Standard Curve method in which target quantity is determined from the standard curve and divided by the target quantity of the calibrator was used.

## Results

### Pathology

**Figure 1:**
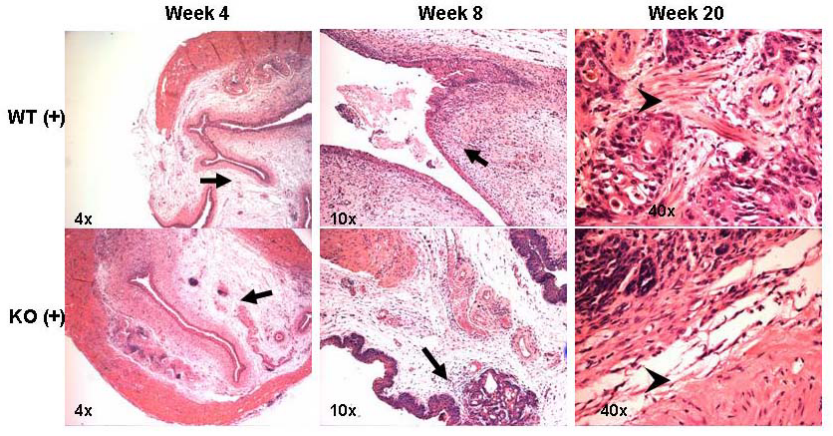
H&E sections from representative time points for MIF WT and KO mice treated with BBN (+). Arrows denote; subepithelial inflammation (4 week) and squamous metaplasia (8 week). High grade carcinoma is present at 20 weeks. Arrowheads show muscle cells with (WT+) and without (KO+) invasive carcinoma.

All BBN treatment groups displayed equal evidence of sub-mucosal inflammation and edema from the earliest time point. Varying degrees of atypia and metaplasia were noted through week 12 (Figure 1). Carcinoma, representing focal areas of CIS, was noted at 16 weeks in both WT and KO mice. At 20 and 23 weeks specimens showed poorly differentiated, high-grade, carcinoma in both genotypes (Figure 1). Tumors were bulky, occupying the majority of bladder lumens precluding precise assessment of the number of tumors present per bladder. However, stage and grade are the most important variables with regard to prognosis in bladder cancer. Although no difference in grade was noted, BBN treated MIF KO bladders (n = 3) had consistently lower tumor stage with no invasion into muscle layers noted (T1). Muscle invasion (T2) was present in all MIF WT bladders (n = 5) at 20–23 wks representing a 100% discordance in phenotype. One MIF KO mouse died prior to sacrifice at 22 weeks with cancer noted but was excluded from analysis because necrolysis prevented accurate tumor staging.

### Immunohistochemistry

**Figure 2:**
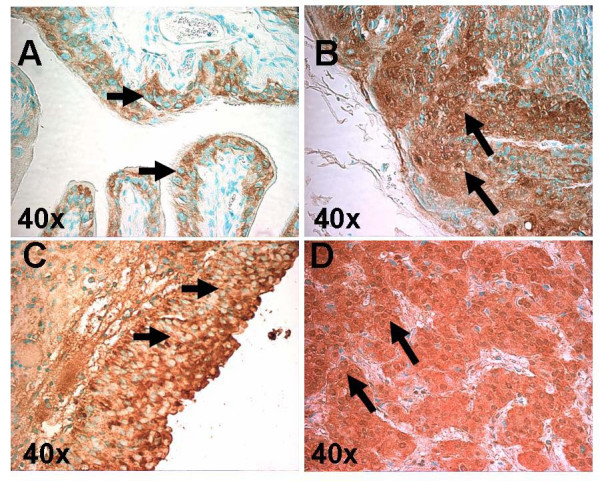
Immunohistochemical staining for MIF in sections from MIF WT control (A), BBN treated mice with bladder cancer at 20 weeks (B) and human specimens from benign (C) and high-grade, invasive TCC (D). Short arrows denote cytoplasmic staining in WT control and benign human tissue (A, C) with relatively scant nuclear staining. Long arrows denote cytoplasmic and intense nuclear staining in murine and human cancer tissue (B, D).

At early time points (4–16 wks) MIF was noted to be constitutively expressed and localized to the urothelial layer in the WT groups, which is in agreement with a prior report [10]. No staining was seen in the KO mice. Staining intensity revealed no pathologic difference between groups treated with and without BBN including areas of metaplasia and CIS. No change in cellular localization was seen either. At 20 weeks when cancer was present, increased intensity of MIF staining was noted in WT treated specimens relative to earlier specimens and to WT untreated specimens. WT control animals showed predominantly cytoplasmic staining with scant nuclear staining confined to the urothelial layer. BBN treated animals showed intense cytoplasmic and nuclear staining in a large proportion of tumor cells (Figure 2). Mean nuclear H-scores for WT control and BBN-treated animals were 63.3 and 152 respectively (p = 0.2, based on the Wilcoxon rank sum test of location difference).

**Figure 3:**
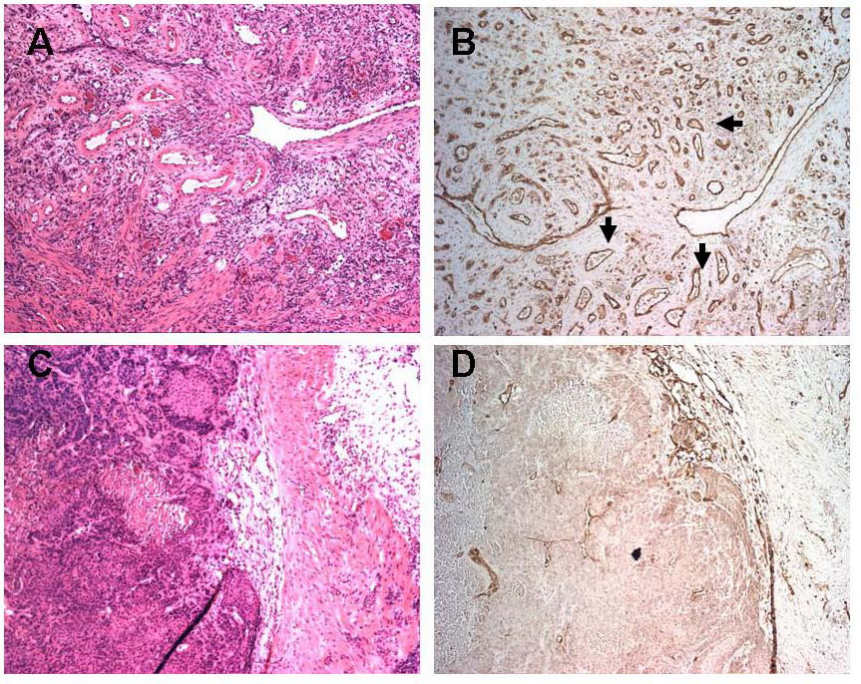
Bladder cancer specimens from MIF WT (A, B) and KO (C, D) animals treated with BBN. A and C are representative areas of tumor with (A) and without (C) muscle invasion. B and D show immunohistochemical staining for vascular structures (arrowheads) by PECAM-1 in the same region. Note the marked increase in number of vessels in the WT animal (B) as compared to the KO (D).

PECAM-1 staining showed an increase in the number of tumor associated vessels in the WT as compared to KO animals (Figure 3). Mean MVD ± SE for the WT and KO specimens were 11.7 × 10^6 ^± 1.7 × 10^6 ^and 6.7 × 10^6 ^± 1.0 × 10^6 ^respectively (p = 0.04, based on the two sample t-test for difference in means).

### mRNA expression

**Figure 4:**
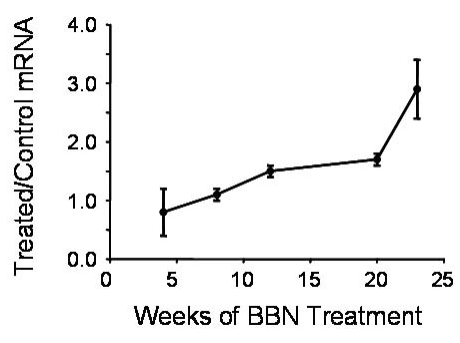
Real-time PCR for MIF mRNA expression in bladders from WT mice. Mice were euthanized after treatment with or without BBN for the weeks indicated. The treated to control ratio was calculated by dividing each RQ (relative quantification) value in the BBN group by the mean of the corresponding control group. Each symbol is the mean ± SD for 2–3 mice per group. All RQ values were calculated after normalization to GAPDH.

MIF mRNA levels were evaluated in bladders from WT animals. The KO bladders had no evidence of MIF mRNA expression by RT-PCR or protein by IHC. MIF mRNA levels gradually increased in the treated animals versus controls (Figure 4). By week 23 when invasive cancer was present, the observed MIF mRNA level was 3-fold higher in the BBN treated mice compared to controls. TNFα, a marker of inflammation, was elevated equally in WT and KO treated animals from the earliest time points.

To investigate the statistical significance of the treatment effect we fit a linear regression model to the data. Residual diagnostics for non-constant variance and non-normality revealed no evidence of model inadequacy. We tested for an overall treatment effect by performing a partial F-test with p < .01, indicating a highly statistically significant overall treatment effect.

## Discussion

MIF is a widely expressed molecule first described for its ability to inhibit the random migration of macrophages in vitro [11]. Its participation in the host response to inflammation and defense is well established [12]. As a result of its biological actions, MIF may prove to play an important role in tumorigenesis. MIF activates the MAPK/ERK signaling pathway, which regulates cellular proliferation and survival. MIF has been reported to inhibit p53 tumor suppressor activity via a COX-2/PGE_2 _dependant pathway resulting in cell growth and preventing cell regulated apoptosis [2,13]. It has also been reported to increase cyclin D transcription leading to increased phosphorylation of pRb resulting in cellular proliferation [3,14]. In addition, MIF has recently been shown to promote survival in fibroblasts via a phosphoinositide-3-kinase (PI3K)/Akt signaling pathway [15]. Recent publications have suggested that MIF expression can lead to increased angiogenesis which is integral to cancer growth, invasion and metastasis [16,17].

MIF has been implicated in prostate, lung and breast cancer with overexpression shown to correlate with tumor grade/stage and prognosis [4-6]. Bladder epithelial cells not only produce MIF but may also display upregulation in response to diverse stimuli [10,18]. Recently, inhibition of MIF with hyaluronic acid, anti-MIF antibody or MIF anti-sense, was shown to decrease in vitro bladder cancer cell proliferation and cytokine expression [10].

Our results suggest that MIF may be involved in the progression of bladder cancer to muscle invasion. The absence of MIF did not impair tumor development but was associated with the absence of tumor invasion into muscle. Muscle invasive disease was present in all WT animals and was associated with a notable increase in stromal angiogenesis. At the time of muscle invasion MIF mRNA levels were three-fold higher in the treated versus control animals. In addition, a pattern of MIF protein nuclear redistribution was noted. mRNA levels for TNFα, another inflammatory mediator, were elevated equally in both the WT and KO mice throughout the duration of the study and pathologic evidence of inflammation (sub-mucosal edema, inflammatory cell infiltrate) was equal as well, suggesting no difference in exposure to metabolites of BBN.

The mechanism by which MIF may facilitate invasion of tumor cells is currently unknown. Cell lines have shown an increased expression of matrix metalloproteases, important in the breakdown of the extracellular matrix, when exposed to recombinant MIF [19,20]. Cancer cells have demonstrated an increase in invasive capacity when exposed to recombinant MIF [16] or co-cultured with macrophages leading to a TNF-α dependant increase in MIF expression via an NFκ-B pathway [17]. Additionally, we have recently reported both *in vitro *and *in vivo *evidence that MIF promotes bladder muscle cell death which could facilitate tumor cell invasion [21].

Angiogenesis is recognized as an important step in tumor invasion and metastasis. Proangiogenic factors have been identified in bladder cancer and MVD has been shown to be a prognostic indicator [22]. Many of the same studies noted above also evaluated angiogenesis and were able to show increased angiogenic properties related to the expression of MIF. Ovarian and breast cancer cells had a MIF-dependent increase in angiogenic factors when co-cultured with macrophages [17]. In vivo studies utilizing small interfering RNA (siRNA) transfected cells and inoculation of nude mice showed tumors with less associated angiogenesis and invasive capacity [16]. Our study compliments these findings in an *in vivo *model by showing a statistically significant increase in tumor associated vascular structures in MIF WT compared to KO mice.

Translocation of protein from the cytoplasm to the nucleus is seen in many regulatory molecules. Proteins reported to do so in cancer may interact with transcription factors important for cell growth, angiogenesis and invasion. Nuclear redistribution of MIF has been seen in tumor tissues from lung adenocarcinoma patients and glioblastoma multiforme tissue [23,24]. Although MIF was seen in normal lung tissue by IHC, staining was noted only in the cytoplasm. As in our study, MIF was seen only in the cytoplasm in normal lung tissue by IHC. In lung cancer specimens MIF staining was noted to be more intense and seen in the nuclei of a large portion of the specimens. Stratification of patient outcome was possible based on the presence of nuclear staining. However, contrary to our findings in mice, nuclear staining portended a better clinical outcome for lung cancer patients [23]. In normal brain tissue no cytoplasmic or nuclear staining was noted for MIF yet brain cancer specimens revealed nuclear localization of MIF. We have preliminary data from human bladder cancer specimens showing similar redistribution of MIF to the nucleus when compared to benign tissue (Figure 2).

This is the first report of the impact of MIF gene deletion on the development and progression of bladder cancer. Our findings are derived from a small pilot study and will need to be confirmed and expanded in larger studies that identify the mechanisms of MIF involvement in the progression to muscle invasion.

## Conclusion

MIF KO mice developed consistently lower stage tumors with less associated angiogenesis in a BBN model of bladder cancer. Tumor presence was associated with nuclear redistribution of MIF. These preliminary data suggest that MIF plays a role in the progression to muscle invasive disease.

## Abbreviations

MIF – Macrophage Migration Inhibitory Factor

WT – Wild Type

KO – Knock Out

BBN – N-butyl-N-(4-hydroxybutyl)-nitrosamine

IHC – Immunohistochemistry

MVD – Microvessel Density

PECAM-1 – Platelet Endothelial Cell Adhesion Molecule

## Competing interests

The author(s) declare that they have no competing interests.

## Authors' contributions

JAT and CP designed the study, and along with GK, coordinated all aspects of the study and drafted the manuscript. OSV was involved in RNA extraction, quantitative PCR and interpretation of data. PH and KC provided pathologic analysis and interpretation of IHC. JT provided statistical analysis. RB and LL provided the founder mice for the MIF KO colony at UCHC and reviewed the manuscript. All authors read and approved the final manuscript.

## Pre-publication history

The pre-publication history for this paper can be accessed here:


